# Circulating tumor cells in peripheral blood as a diagnostic biomarker of breast cancer: A meta-analysis

**DOI:** 10.3389/fonc.2023.1103146

**Published:** 2023-03-22

**Authors:** Tao Jin, Yao Chen, Qing-Yan Chen, Yang Xiong, Ji-Qiao Yang

**Affiliations:** ^1^ Department of Gastrointestinal Surgery, West China Hospital, Sichuan University, Chengdu, China; ^2^ Laboratory of Gastric Cancer, State Key Laboratory of Biotherapy/Collaborative Innovation Center of Biotherapy and Cancer Center, West China Hospital, Sichuan University, Chengdu, China; ^3^ Breast Center, West China Hospital, Sichuan University, Chengdu, China; ^4^ Medical college, Hebei University of Engineering, Hebei, China; ^5^ Department of Urology, West China Hospital, Sichuan University, Chengdu, China

**Keywords:** breast cancer, diagnosis, circulating tumor cells, CTCs, meta - analysis

## Abstract

**Purpose:**

Studies have reported that breast cancer (BC) patients’ circulating tumor cells (CTCs) have varying results for their diagnostic role. Thus, we conducted a meta-analysis to systematically assess the accuracy of CTCs in the diagnosis of BC.

**Methods:**

A meta-analysis was conducted to evaluate the overall accuracy of CTC detection. A pooled analysis of sensitivity (SEN), specificity (SPE), positive likelihood ratio (PLR), negative likelihood ratio (NLR), and diagnostic advantage ratio (DOR) was used to measure diagnostic accuracy. In addition, the area under the summary receiver operating characteristic curve (AUC) was used to discriminate BC from non-BC. An analysis of the threshold effect was calculated using the Spearman correlation coefficient. We calculated the Q and I2 statistics to determine whether the studies were heterogeneous. Sensitivity analysis was performed by removing studies one by one. Publication bias was assessed by Deeks’ funnel plot asymmetry test.

**Results:**

Studies from the PubMed, Cochrane Library, Embase, Web of Science, Wanfang, Vip, and CNKI databases were collected for diagnosing BC from January 2000 to April March 2023. Finally, 8 publications were retrieved in total containing 2014 cases involved in the study. Based on a random-effects model, it was found that the pooled SEN was 0.69 (0.55 - 0.80), SPE was 0.93 (0.60 - 0.99), PLR was 9.5 (1.4 - 65.9), NLR was 0.33 (0.23 - 0.48), DOR was 29 (4 - 205) and the AUC of the summary receiver operating characteristic (SROC) curve was 0.81 (0.77 - 0.84). Some heterogeneity was found in the article, but there was no threshold effect to account for it (P = 0.27). Deek’s funnel plot asymmetry test indicated that no publication bias was observed in this meta-analysis (P = 0.52).

**Conclusion:**

The results of this meta-analysis confirmed that CTCs were an important component of noninvasive methods of confirming BC with SEN of 0.69 (0.55 - 0.80), SPE of 0.93 (0.60 - 0.99) and AUC of 0.81 (0.77 - 0.84).

## Background

Among women, BC is the most prevalent cancer and is influenced by lifestyle factors, hormonal factors, reproductive factors, and iatrogenic factors. Furthermore, recent data from 185 countries reported 2.3 million new cases (more than 10% of all cancers) of breast cancer and a mortality rate of 6.9%, and BC ranked the second leading cause of death from cancer among women globally (1, 2). During the past few decades, the morbidity of BC has continued to increase around the world (3). Furthermore, a study showed a significant increase in breast cancer mortality rate in low-income regions, while the decreasing rate mostly belongs to Western Europe, with 37.57 in 1990 to 36.00 in 2015 (4). Due to advances in screening methods and breakthroughs in early diagnosis and treatment, BC survival rates have improved. The conventional diagnostic methodologies of BC include breast biopsy, which is regarded as the gold standard, and imaging methods without high sensitivity to detect BC in the early stage (5). In addition, molecular markers, including CA15-3 and CEA, are common markers for monitoring and follow-up of patients by testing BC patient blood samples, but they have low SEN and SPE (6–8). Thus, it was not suitable for the detection of BC. To improve BC cure rates and reduce BC mortality, early diagnosis remains essential. Thus, it is necessary to explore a new test with high SEN and SPE to diagnose BC in the early stage. Recently, a hot research topic about tumors has been the clinical application of CTCs. CTCs, a subset of tumor cells that circulate within the body due to tumor tissue instability or external physical stimulation, participate in the body’s circulation and then integrate into the peripheral blood circulation (9, 10). The Fourth Edition of the National Comprehensive Cancer Network (NCCN) guidelines has added a new M0 (i+) category, which is defined as “no clinical or radiographic evidence of distant metastases, but the presence of detected tumor cells in the circulation fluids” (11). In addition, CTCs have demonstrated efficacy in the screening of malignant cancers such as prostate, lung, and colorectal cancers (9). In a recent study, CTCs were found to be 76.56% sensitive and 95.4% specific for diagnosing breast cancer using the CytoSorter^®^ (12). Nonetheless, several studies have been conducted on CTCs to diagnose BC with varied results by testing peripheral blood. In addition, current studies have shown that CTC detection positive rates (≥ 1 CTC/7.5 ml) range from 11%~54% for early breast cancer, while ≥1 CTC can be detected in approximately 70% of stage IV BC patients. Thus, a meta-analysis was performed to determine whether CTCs are particularly useful as a diagnostic tool in patients with BC.

## Methods

This study was conducted according to the Preferred Reporting Items for Systematic Reviews and Meta-analyses (PRISMA) guidelines (13).

### Literature search

We conducted a comprehensive computer literature search of abstracts from human studies to identify articles about the effectiveness of CTC tests for diagnosing BC by two independent individuals (Tao Jin and Yao Chen). Electronic databases such as PubMed, Embase, Cochrane Library, Web of Science, Wanfang, CNKI, and Vip were used with the following search terms: “CTCs”, “circulating tumor cells”, “breast cancer”, “breast carcinoma”, “accuracy”, “sensitivity and specificity”, from January 2000 to March 2023, without language limitation. We manually searched references in the included literature to identify studies that met our eligibility criteria, and gray literature was also included in the study.

### Literature eligibility

The included studies were screened according to the following criteria: (1) Type of trial: studies applying the method of detecting CTCs to diagnose breast cancer; (2) Diagnostic gold standard: histopathological examination or biopsy results; (3) The literature should include sufficient study data including true positive (TP), false positive (FP), true negative (TN), false negative (FN); (4) patients without other malignant tumors; (5) we chose the article with the most detail or the most recent when more than one article presented the same data or subset of data. Exclusion criteria were (1) insufficient information in the literature to obtain complete diagnostic data from the full text of the literature and (2) reports on cases, reviews, letters, single-arm trials, editorials, and duplicate studies.

### Data extraction and quality assessment

Two independent researchers (Tao Jin and Yao Chen) reviewed all studies. Disagreements between researchers were resolved through discussion and consensus. In the case of disputes, an independent third researcher was responsible for resolving disagreements. The main data information included author, year of publication, country, tumor stage, isolation enrichment method, assay identification method, CTC cutoff, TP, FP, FN, and TN. Data for results not directly reported were derived from estimates of SEN and SPE, along with positive and negative predictive values. Primary outcome measures were pooled estimates of SEN and SPE. Evaluation of the quality of the included literature was carefully conducted using the Quality Assessment of Diagnostic Accuracy Studies-2 (QUADAS-2) (14) by two independent reviewers. The inconsistent evaluation was decided by discussion.

### Statistical analysis

The diagnostic accuracy of CTC detection in BC was determined using Stata (version 15.0). Pooled analysis of SEN, SPE, PLR, NLR, and DOR and the corresponding 95% confidence interval (CI) was used to evaluate diagnostic accuracy. The SROC was performed using a bivariate regression approach to identify abnormal examinations that resulted in the expected trade-off between SEN and SPE. In addition, the AUC can summarize the inherent capacity of a test for discriminating BC from non-BC. The threshold effect was analyzed using Spearman correlation coefficients in the heterogeneity analysis. The heterogeneity of the studies was evaluated by the Q test and I2 statistics. I^2^ values ≥50% indicated substantial heterogeneity; additionally, we considered the difference to be statistically significant at P < 0.05. Sensitivity analysis was performed by a one-by-one exclusion method to determine whether the hypothesis had a significant effect on the results. Deeks’ funnel plot asymmetry was used to assess publication bias, and a significance level of P < 0.05 was considered significant.

## Results

### Literature search results

**Figure 1:**
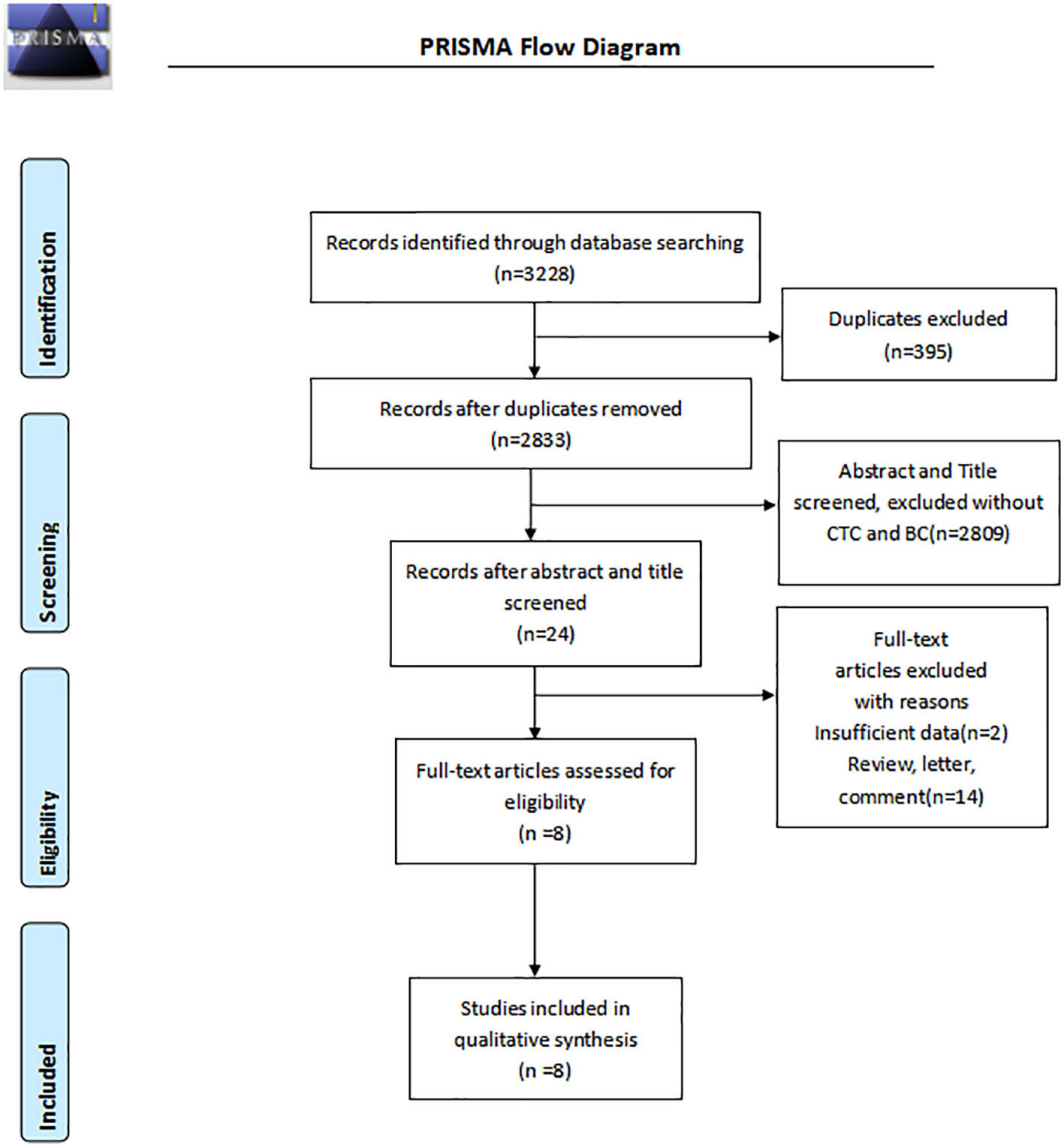
A flow chart of the search and selection of relevant studies.

A total of 3225 pieces of literature were retrieved through electronic databases. After excluding duplicates and irrelevant studies, we carefully and independently reviewed the titles and abstracts. Finally, eight studies (12, 15–21), including 2014 cases, met the requirements through careful screening by two independent researchers after reading the full text in detail. The flow diagram in **Figure 1** illustrates the process of searching for eligible studies.

### Basic characteristics and quality assessment

A summary of the basic characteristics of the included studies is provided in **Table 1**. All patients were diagnosed with stage I to IV disease. Seven studies were from Asia, and one study was from Western countries. Four, three, and one articles set the CTC cutoff as 2, 1, and 1.5, respectively. The enrichment methods of CTCs included negative enrichment, density gradient centrifugation, CytoSorter, immunomagnetic bead, and CellSearch. Most of the articles used imFISH to identify CTCs. **Table 2** presents the results of the QUADAS-2 assessment. Patient selection and index tests accounted for the majority of bias risks.

**Table 1:** Main characteristics of studies included in the meta-analysis of the diagnostic accuracy of CTCs detection in BC.

Author	Year	Country	Stage	Enrichment method	Identification method	CTC cut-off	TP	FP	FN	TN
Qiu	2020	China	I-IV	Negative enrichment	ImFISH	2	105	2	34	346
Wang	2020	China	I-IV	Density gradient centrifugation	ImFISH	2	102	59	27	4
Ji	2020	China	I-IV	Density gradient centrifugation	Nucleic acid testing	1.5	23	0	37	50
Wang	2018	China	I-IV	Immunomagnetic bead	ImFISH	1	25	0	20	10
Gao	2021	China	I-IV	CytoSorter	ImFISH	2	199	76	39	161
Jin	2019	China	I-IV	CytoSorter	ImFISH	2	109	38	19	223
Murray	2015	Chile	NA	Density gradient centrifugation	ImFISH	1	58	6	20	60
Xue	2021	China	I-IV	CellSearch	CellSearch	1	16	18	26	102

**Table 2:** The results of quality assessment of included studies in the meta-analysis.

Study	Risk of Bias				Applicability Concerns		
	Patient Selection	Index Test	Reference Standard	Flow and Timing	Patient Selection	Index Test	Reference Standard
Qiu 2020	low	unclear	low	low	low	low	low
Wang 2020	low	unclear	low	low	low	low	low
Ji 2020	high	unclear	low	low	low	low	low
Wang 2018	high	unclear	low	low	high	low	low
Gao 2021	high	high	low	low	high	low	low
Jin 2019	low	high	low	low	high	low	low
Murray 2015	low	unclear	low	low	low	low	low
Xue 2021	low	unclear	low	low	low	low	low

### Accuracy of CTCs in the diagnosis of BC

**Figure 2:**
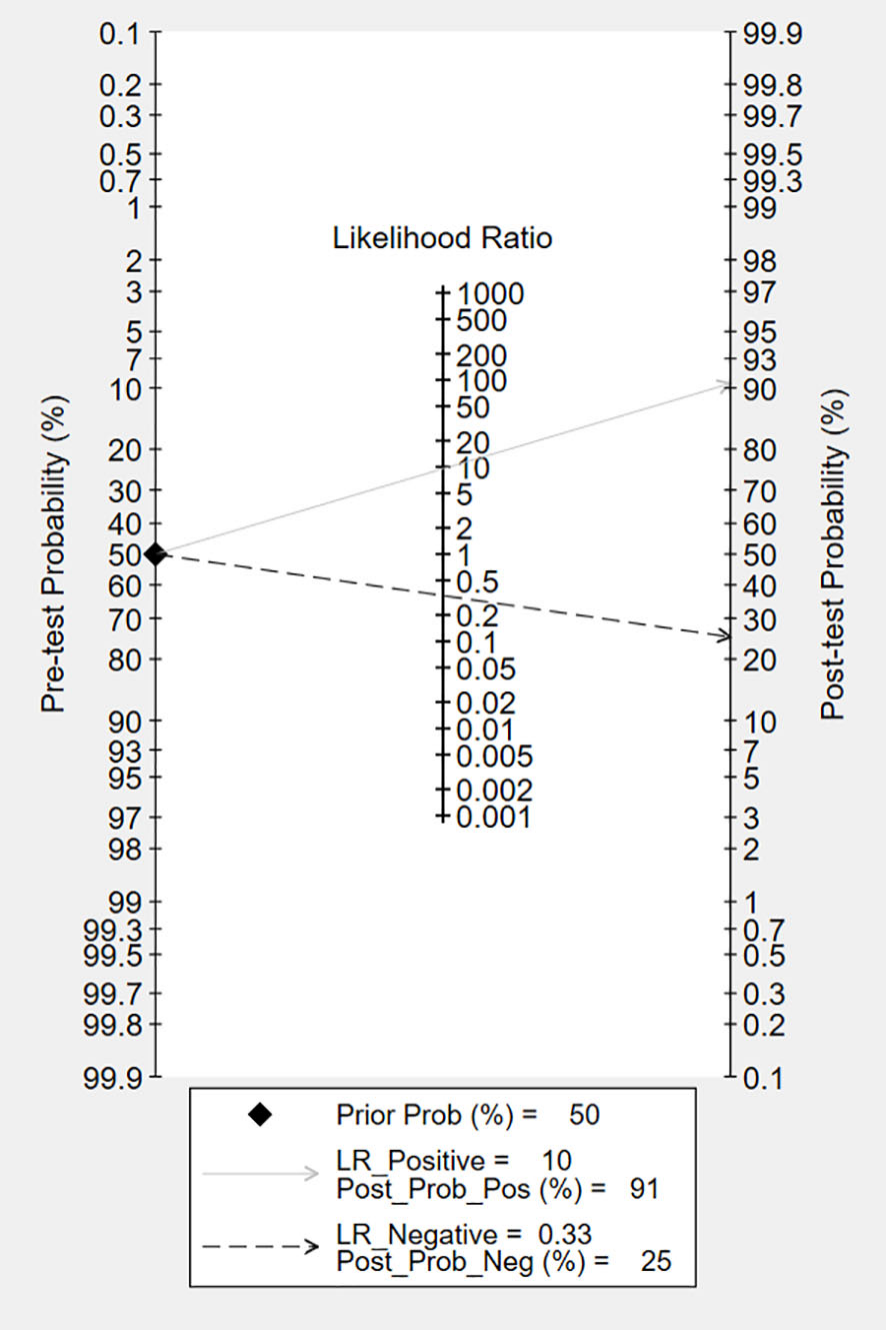
Forest plots depicting the SEN and SPE of CTCs in BC diagnosis.

**Figure 3:**
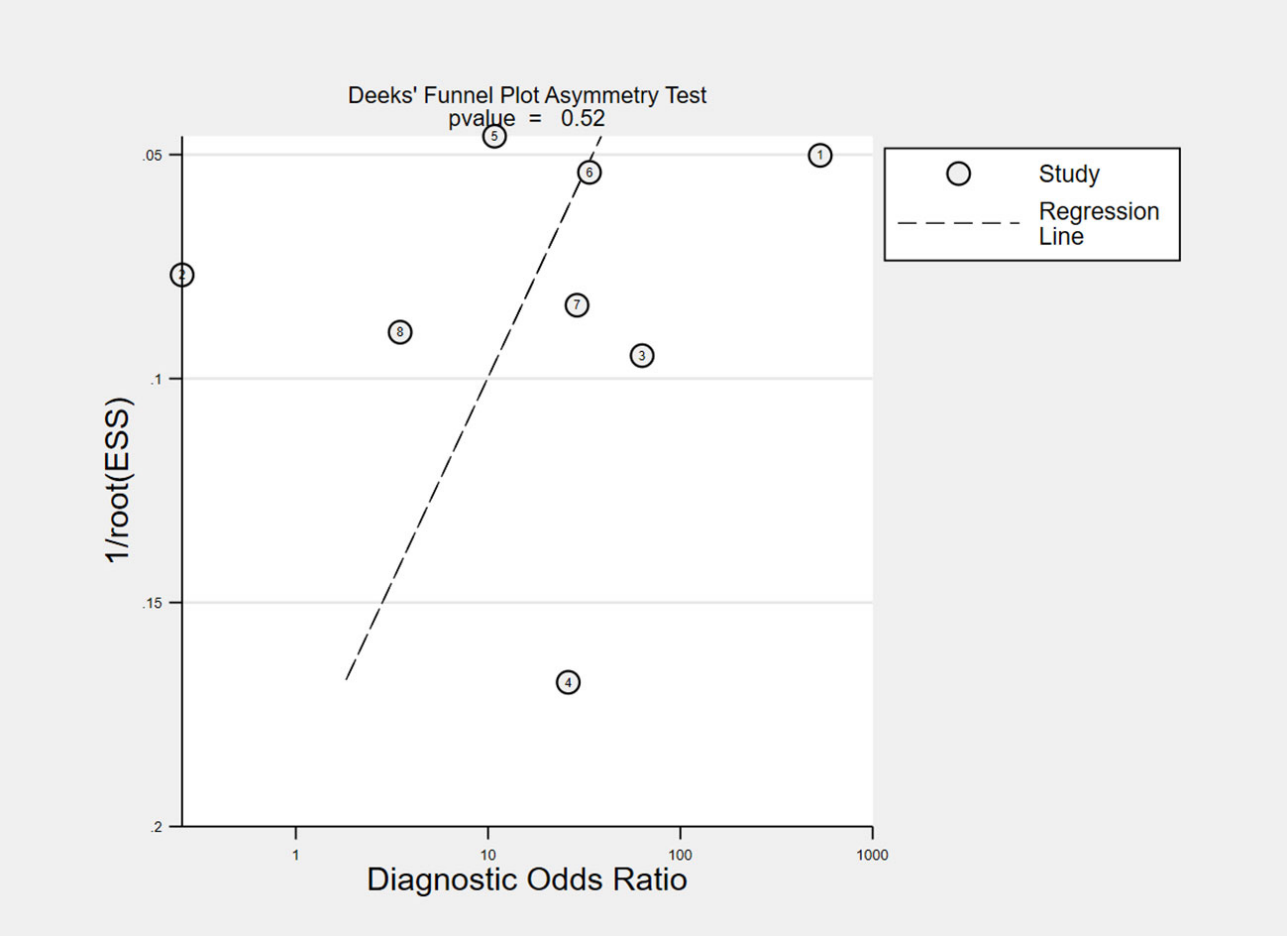
SROC of CTCs in the diagnosis of BC.

**Figure 4:**
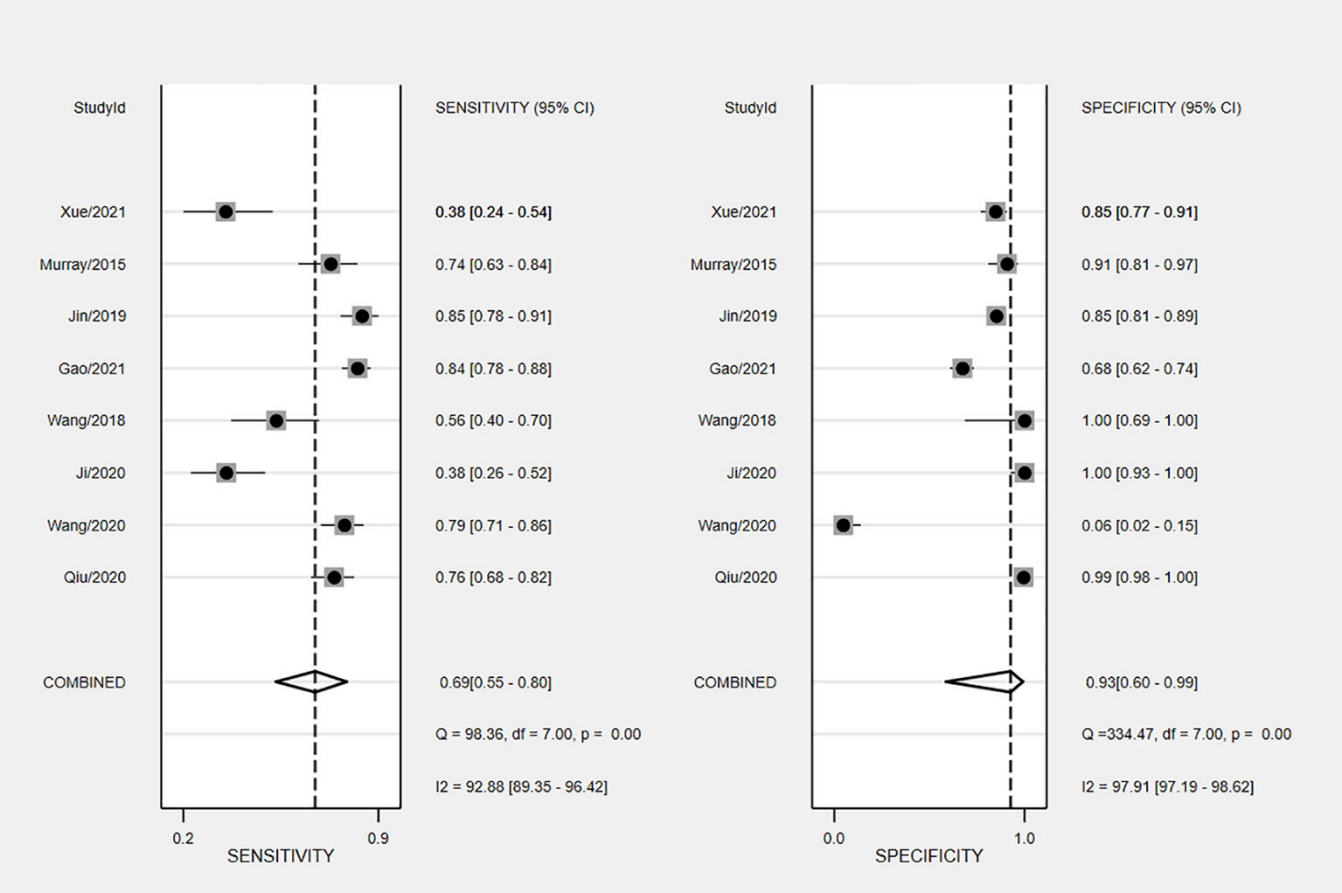
Fagan nomogram plot analysis for the evaluation of CTCs as a diagnostic tool for detecting BC.

**Figure 5:**
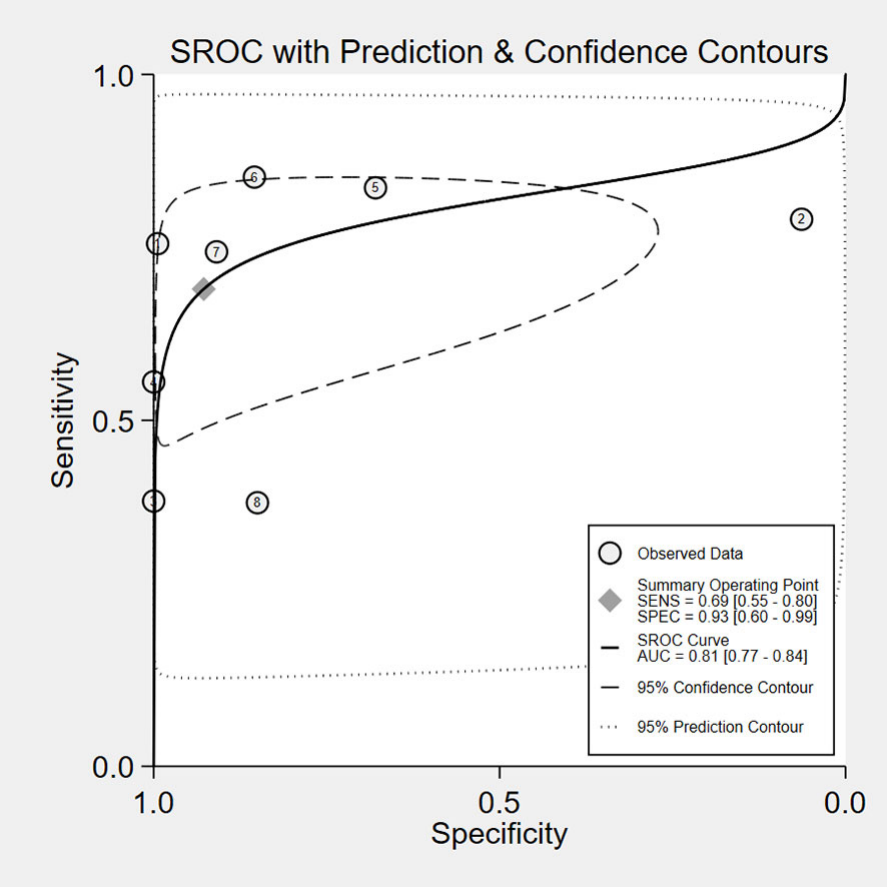
Deeks’ funnel plot for detecting publication bias. ESS, effective sample size.

The overall accuracy of CTCs in diagnosing BC was as follows: SEN, 0.69 (0.55 - 0.80); SPE, 0.93 (0.60 - 0.99) (**Figure 2**); PLR, 9.5 (1.4 - 65.9); NLR, 0.33 (0.23 - 0.48); and DOR, 29 (4 - 205). **Figure 3** shows the SROC plot with a 95% CI. The AUC for BC was 0.81 (0.77 - 0.84). The percentage of heterogeneity caused by the threshold effect was 0.27, while the coefficient of correlation in the mixed model was -0.52, which meant no significant influence of the threshold effect. **Figure 4** presents the Fagan plot, showing that the prior-test probability of BC was 50%. Furthermore, the posttest probability of BC, given a negative result, was 25%, while 91% had a positive result for CTC detection in this meta-analysis. Deek’s funnel plot asymmetry test demonstrated that the slope coefficient P value was 0.52, suggesting that there was no significant publication bias (**Figure 5**). Sensitivity analysis (**Table 3**) showed a slight change when removing articles one by one, indicating that the results were robust.

**Table 3:** Meta-analysis sensitivity analysis for included studies.

Without study	Heterogeneity (*I*2,%)	Sensitivity (95%CI)	Heterogeneity (*I*2,%)	Specificity (95%CI)
None	92.88	0.69 [0.55-0.80]	97.91	0.93[0.60-0.99]
Qiu 2020	93.83	0.68 [0.52-0.80]	97.07	0.88 [0.48-0.98]
Wang 2020	93.46	0.67 [0.52-0.80]	95.14	0.95 [0.81-0.99]
Ji 2020	90.18	0.73 [0.61-0.82]	98.1	0.87 [0.51-0.98]
Wang 2018	93.66	0.71 [0.56-0.82]	98.22	0.90 [0.51-0.99]
Gao 2021	92.04	0.66 [0.52-0.78]	98.26	0.95 [0.59-1.00]
Jin 2019	92.93	0.66 [0.52-0.78]	98.13	0.94 [0.54-1.00]
Murray 2015	93.86	0.68 [0.52-0.80]	98.13	0.93 [0.52-0.99]
Xue 2021	91.80	0.73 [0.68-0.82]	98.15	0.97 [0.41-1.00]

## Discussion

Breast ultrasound and mammography are currently the main methods for screening BC, but with low SEN, they are easily influenced by breast density, and the incidence of false negatives and false positives is high (22). Serological markers such as carbohydrate antigen CA153 and carcinoembryonic antigen (CEA) have the characteristics of noninvasiveness, nonradiation, and low price but still have low SEN and SPE. Thus, they are not suitable for the early diagnosis of BC (23). CTCs are cancer cells that contain unique biomarkers and are commonly found in blood samples from individuals with solid tumors but often not in healthy populations. The prognostic relevance of CTCs in many types of metastatic cancer has already been demonstrated (24–26). According to the eighth AJCC cancer staging manual, BC patients with CTCs are at a greater risk for poor outcomes (27). In recent years, CTC detection has been proven to be helpful in the diagnosis of lung cancer (28), bladder cancer, urothelial cancer (29), pancreatic cancer (30), and so on. Furthermore, CTCs in peripheral blood have been used to diagnose BC in a limited number of studies, with varying results. Consequently, we conducted the first meta-analysis to assess the diagnostic value of CTC detection in the peripheral blood of BC patients.

The results of the study, including 2014 individuals from 8 diagnostic accuracy studies, proved that CTCs had high clinical utility in the diagnosis of breast cancer, with a pooled SEN of 0.69 (0.55-0.80), a pooled SPE of 0.93 (0.60-0.99), a pooled LNR of 9.5 (1.4-65.9), a pooled NLR of 0.33 (0.23-0.48), and a total AUC of the SROC curve of 0.81. These results show that the overall accuracy of CTCS in the early diagnosis of BC is relatively good. After sensitivity analysis, the results of the literature we included were stable, indicating that our meta-analysis results are of reference significance. By using the DOR, a diagnostic test evaluation indicator, we could compare the likelihood of positive results between patients with and without the condition. In the present analysis, the pooled DOR was 29 (95% CI, 4–205), indicating that in comparison to patients who do not test positive for CTCs, those who test positive have 29 times the likelihood of developing BC. Based on the above results, CTCs might be helpful as a diagnostic method for BC screening, which is in accordance with a prior study (12). However, the included studies have different CTC detection methodologies, as well as different sensitivity levels, resulting in a varying CTC cutoff value for the same clinical application (31). To date, CellSearch^®^ has been the only CTC system approved by the Food and Drug Administration (FDA). However, CellSearch^®^ had low rates of CTC detection in BC, approximately 40–50% in metastatic BC and just under 30% in early-stage BC (32). There was only one study (21) using CellSearch^®^ in our meta-analysis. Another study (12) reported the CytoSorter^®^ CTC detection system. CytoSorter^®^ was shown to be superior to CellSearch^®^ in detecting CTCs in BC patients at stages II and III, with a detection rate of over 90% (32). Due to the lack of uniform detection standards for CTCs, clinical practice does not consider CTCs to be a standard routine diagnostic tool. Thus, more research is required to determine the criteria for CTC detection. Based on the use of different threshold values in the included studies, we used Spearman’s correlation coefficient to analyze threshold effects and found that there was no connection between thresholds and heterogeneity.

This study has some limitations. First, on account of the relatively small number of cases in this study, we failed to determine the potential source of this study due to the relatively high heterogeneity of this study. Second, in various studies, cutoff values differ, which has an impact on our results, and there is a need for further research on CTCS’s optimal cutoff point. In addition, seven out of eight studies were conducted in Asia, and the electronic databases included regional databases, which could cause bias in the results. It would be beneficial to conduct more international prospective multicenter research on this topic.

## Conclusion

This meta-analysis showed that CTCs can be used as a helpful tool in BC screening and early diagnosis, with better sensitivity and specificity. To clarify the accuracy of CTCs as BC diagnostic indicators, more high-quality prospective studies are needed.

## Data availability statement

The original contributions presented in the study are included in the article/supplementary material. Further inquiries can be directed to the corresponding author.

## Author contributions

All authors contributed to the study conception and design. Material preparation, data collection and analysis were performed by TJ, YC and J-QY. The first draft of the manuscript was written by TJ, and all authors commented on previous versions of the manuscript. All authors contributed to the article and approved the submitted version.
